# ADSC-derived exosomes attenuate myocardial infarction injury by promoting miR-205-mediated cardiac angiogenesis

**DOI:** 10.1186/s13062-023-00361-1

**Published:** 2023-02-27

**Authors:** Tingting Wang, Tao Li, Xiaolin Niu, Lang Hu, Jin Cheng, Dong Guo, He Ren, Ran Zhao, Zhaole Ji, Pengyun Liu, Yan Li, Yanjie Guo

**Affiliations:** 1 Department of Cardiology, Tangdu Hospital, Fourth Military Medical University, Xi’an, 710032 China; 2 Heart Hospital, Xi’an International Medical Center, Xi’an, 710038 China; 3 Fourth Military Medical University, Xi’an, 710032 China; 4 Ultrasound Diagnostic and Treatment Center, Xijing Hospital, Fourth Military Medical University, Xi’an, 710038 China

**Keywords:** Myocardial-infarction, Adipose-derived mesenchymal stem cells, Exosomes, miRNA-205, Angiogenesis

## Abstract

**Background:**

Acute myocardial infarction is a major health problem and is the leading cause of death worldwide. Myocardial apoptosis induced by myocardial infarction injury is involved in the pathophysiology of heart failure. Therapeutic stem cell therapy has the potential to be an effective and favorable treatment for ischemic heart disease. Exosomes derived from stem cells have been shown to effectively repair MI injury-induced cardiomyocyte damage. However, the cardioprotective benefits of adipose tissue-derived mesenchymal stem cell (ADSC)-Exos remain unknown. This study aimed to investigate the protective effects of exosomes from ADSC on the hearts of MI-treated mice and to explore the underlying mechanisms.

**Methods:**

Cellular and molecular mechanisms were investigated using cultured ADSCs. On C57BL/6J mice, we performed myocardial MI or sham operations and assessed cardiac function, fibrosis, and angiogenesis 4 weeks later. Mice were intramyocardially injected with ADSC-Exos or vehicle-treated ADSCs after 25 min following the MI operation.

**Results:**

Echocardiographic experiments showed that ADSC-Exos could significantly improve left ventricular ejection fraction, whereas ADSC-Exos administration could significantly alleviate MI-induced cardiac fibrosis. Additionally, ADSC-Exos treatment has been shown to reduce cardiomyocyte apoptosis while increasing angiogenesis. Molecular experiments found that exosomes extracted from ADSCs can promote the proliferation and migration of microvascular endothelial cells, facilitate angiogenesis, and inhibit cardiomyocytes apoptosis through miRNA-205. We then transferred isolated exosomes from ADSCs into MI-induced mice and observed decreased cardiac fibrosis, increased angiogenesis, and improved cardiac function. We also observed increased apoptosis and decreased expression of hypoxia-inducible factor-1α and vascular endothelial growth factor in HMEC-1 transfected with a miRNA-205 inhibitor.

**Conclusion:**

In summary, these findings show that ADSC-Exos can alleviate cardiac injury and promote cardiac function recovery in MI-treated mice via the miRNA-205 signaling pathway. ADSC-Exos containing miRNA205 have a promising therapeutic potential in MI-induced cardiac injury.

**Supplementary Information:**

The online version contains supplementary material available at 10.1186/s13062-023-00361-1.

## Background

Acute myocardial infarction (AMI) is the leading cause of death in both developed and developing countries [1]. The adoption of optimal primary percutaneous coronary intervention and early thrombus treatment has recently resulted in the reduced size of myocardial infarction areas and fewer MI-related deaths [2, [3]. However, myocardial infarction (MI) injury, which includes increased oxidative stress and inflammation, still induces acute or chronic loss of cardiomyocytes, eventually leading to impaired cardiac function and heart failure [2, [4]. Stem cell therapy for the regeneration of damaged cardiomyocytes has received much attention recently, and diverse cell types have been employed in stem cell therapy**.** Adipose-derived stem cells (ADSCs) are more easily and abundantly obtained by minimally invasive procedures than bone marrow mesenchymal stem cells (MSCs) [5]. Apart from this, ADSCs have been shown to play a vital role in repairing damaged cardiomyocytes, making them the most promising therapeutic candidate [6].

ADSCs administration has been shown to reduce myocardial infarct size and improve cardiac function [4]. Currently, ADSC-based therapy for infarcted myocardium mainly comprises intracoronary or trans-endocardial injection, direct intramyocardial injection, and other invasive techniques [2, [7]. Various studies have shown that the invasive injection of ADSCs results in limited retention of stem cells in the myocardium, limiting the beneficial effects on infarcted cardiomyocytes [8]. Although the protective effect of ADSC-based therapy in the myocardium after cardiac MI injury has been widely reported, the underlying mechanism by which ADSCs improve cardiac injury after MI remains unclear. Exosomes are membrane lipid vesicles with a diameter of 30–100 nm. Exosomes secreted by stem cells are the most effective paracrine components for active cell-to-cell communication, and they show remarkable therapeutic potential for repairing damaged tissue [6, [9, [10]. Moreover, it has been suggested that exosomes derived from stem cells can effectively repair damaged cardiomyocytes after MI injury [9, [11]. Therefore, ADSCs and ADSC-Exos have potential clinical applications.

Exosomes contain a diverse range of biomolecules, including DNA, mRNAs, miRNAs, proteins, and lipids, with miRNAs being the most abundant [12]. MiRNAs are small (∼22 nucleotides) non-coding RNAs [6] that are involved in cell differentiation, proliferation, and apoptosis [13]. They have been shown to regulate the expressions of multiple mRNAs and contribute to intracellular communication. They also play an important role in the progression of some diseases such as immune system modulation and metastasis progression in cancer [14]. Recent studies have shown that miRNA expression is associated with cardiac events, and changes in miRNA expression can give rise to heart diseases such as MI and heart failure [15, [16]. MiRNAs in exosomes may have therapeutic effects in MI injury. Previous studies have demonstrated that miRNA-205 regulation can inhibit apoptosis [17]. Various myocardial cell injuries can cause apoptosis, which can lead to heart failure [18]. Thus, miRNA-205 is a potential therapeutic target to reduce myocardial damage through the inhibition of myocardial apoptosis.

This study aimed to investigate the protective effects of ADSC-Exos on the hearts of MI mice and to explore the underlying mechanisms. Our results indicate that ADSC-exos attenuated cardiac injury and promoted cardiac functional recovery. We also found that ADSC-exos can promote microvascular endothelial cell proliferation, facilitate angiogenesis, and inhibit cardiomyocyte apoptosis through the miRNA-205 signaling pathway. In summary, our data provide strong evidence that ADSC-Exos containing miRNA205 is beneficial for MI injury and has clinical applications.

## Materials and methods

### Mouse MI model

Male mice were anesthetized with isoflurane (1–2%) (8–12 weeks), the hearts of the mouse were rapidly squeezed out of the chest cavity through the left thoracic incision. In order to induce myocardial infarction, we used silk thread (6–0) to ligate the left anterior descending (LAD) coronary artery. Whitening of ischemic area and changes of ECG are important indicators of successful operation. The sham operated control mice received the same procedure without coronary artery ligation [19].

### Animal study protocol

We purchased 30 8–12 weeks old male C57BL/6 wild-type mice (body weight: 25–30 gm) from the Laboratory Animal Center of the Fourth Military Medical University. The mice were anesthetized on a C57BL/6 background with 2% isoflurane. After 25 min, ADSC-Exos (100 μg protein, 50 μL) was administered evenly intramuscularly into five locations along the anterior wall of the left ventricle’s border zone. The slipknot was released after 40 min to reperfuse the myocardium. All animal experiments were approved by the Animal Care and Use Committee of the Fourth Military Medical University and followed the National Institutes of Health guidelines for the use of laboratory animals (National Institutes of Health Pub. No. 85–23, Revised 2011). The hearts were collected after 4 weeks and fixed with paraformaldehyde or further analysis.

### Isolation of neonatal rat cardiomyocytes (NRCMs) and detection of apoptotic cardiomyocytes

NRCM was isolated from C57BL/6 wild-type mice 1–2 days old. Simply, the heart tissue was washed with PBS three times to remove blood. Then, the hearts were cut into small pieces and digested with type I collagenase solution (1 mg/ml, Thermo Fisher Scientific, Waltham, MA, USA) for 5 to 6 times. Finally, complete medium was added to terminate the digestion process. Because the attachment time of cardiomyocytes was different from fibroblasts, the differential attachment method was used to remove fibroblasts as much as possible. The isolated primary cardiomyocytes were cultured in normal medium for 48 h. Before inducing hypoxia in cardiomyocytes, the medium was replaced with a sugar-free and serum-free medium to simulate nutrient deprivation. Moreover, the cardiomyocytes were placed in a hypoxic chamber (1% O2, 5% CO2, and 94% N2.) for further culturing 9 h. The apoptotis of cardiomyocytes was evaluated by flow cytometry, the images were analyzed by image J software [19].

### ADSC preparation

ADSCs were extracted according to the method described previously [2]. Under anesthesia, mice inguinal subcutaneous fat was harvested. The adipose tissue was washed several times with sterile phosphate-buffered saline (PBS, Sigma) and then the blood vessels in the adipose tissue were removed with the aid of a dissecting microscope. The remaining adipose tissue was digested with 0.1% type I collagenase (catalog number 17018029, ThermoFisher Scientific, USA) at 37 °C for 60 min and then centrifuged at 1000 g for 10 min, non-adherent cells were removed 48 h after the cells were plated. Then, ADSCs were cultured in Dulbecco’s Modified Eagle Medium (DMEM, Gibco) containing 20% Fetal Bovine Serum (FBS) and penicillin/streptomycin at 37 °C in a humidified atmosphere containing 5% CO_2_. Cells from passage 2 were used for all experiments.

### Cell culture

Microvascular endothelial cells were purchased from the American Type Culture Collection (ATCC). Cell culture medium contains DMEM medium (Life Technologies, Grand Island, NY, USA) and 10% heat-inactivated fetal bovine serum (Hyclone, UT, USA). All the cells were incubated in a 37 °C humidified incubator containing 5% CO2.

Before induction of hypoxia in HMEC-1 cells, the medium was replaced with a sugar-free and serum-free medium to simulate nutrient deprivation. In addition, the HMEC-1 cells were placed in a hypoxic chamber (1% O2, 5% CO2, and 94% N2) for further 2 h culture [20].

### Echocardiography

Cardiac function of the mice subjected to I/R after 6 h and 4 weeks were evaluated by echocardiography, as previously described [2]. Mice were anesthetized by inhalation of 1–2% isoflurane, and transthoracic two-dimensional exercise mode echocardiography (VisualSonics) was performed. The Vevo770 software program (VisualSonics) was used to collect and analyze LV end-systolic dimensions (LVESD), LV end-diastolic dimensions (LVEDD) and LV ejection fraction (LVEF) parameters.

### HE and masson staining

Mice were executed and the hearts were isolated. For histological analysis of angiogenesis and fibrosis, HE and Masson staining were performed, a total of 10 Sects. (7–10 um thick) per heart were prepared [2]. HE staining of the sections were performed according to the manufacturer's instruction. Masson’s trichrome was used to evaluate fibrosis in post-I/R mice hearts. The percentage of fibrotic area to total heart represents myocardium fibrosis in post-MI mice hearts. Myocardial fibrosis was quantified by means of Image-Pro plus 6.0 software (Media Cybernetics).

### Immunofluorescence staining

All the sections were blocked with 1% goat serum albumin for 1 h and then incubated with mouse monoclonal anti-CD31 primary antibody (Ab955, 1:200; Abcam) at 4 °C overnight. The sections were then stained with rabbit anti-mouse secondary antibody (1:1000; Abcam) for 1 h at room temperature. The tissue slices were washed and mounted with medium containing DAPI. All slices were observed by the Olympus FV1000 laser confocal microscope.

### Western blot analysis

Protein was extracted from heart or cultured microvascular endothelial cells and ADSCs according to standard Invitrogen protocols (Invitrogen, Carlsbad, CA, USA) as previously described [2]. Protein quantitation was modified by Bradford assay (Bio-Rad Laboratories, Hercules, CA, USA) and then separated by SDSPAGE with primary antibodies against The blots were incubated with primary antibodies as follows: HIF-1α(ab179483; Abcam), VEGF (ab32152; Abcam), CD63 (ab217345; Abcam), CD9 (ab223052; Abcam), TSG101 (ab235011; Abcam), β-actin (ab8226;Abcam), Caspase3 (ab184787; Abcam) overnight at 4 °C. The blots were visualized using a chemiluminescence system (Amersham Bioscience, Buchinghamshire, UK). Anti-β actin antibody (Proteintech, IL, United States) was used as a loading control. The signals were quantified by Image J software.

### Extraction and characterization of ADSC-exos

Cultured ADSCs were plated at 5 × 10^5^ cells in a 10-cm dish. The culture medium was collected and centrifuged at 13,000 g for 30 min to remove cells and cell debris. According to the manufacturer's instructions (System Biosciences, CA, United States), 2 mL of ExoQuick-TCTM exosome precipitation solution was used to isolate ADSC-Exo from 10 mL of culture medium. After overnight incubation at 4 °C, the mixture was centrifuged at 10,000 g for 30 min at 4 °C. After being washed, the exosomes were centrifuged at 10,000 g for 15 min at 4 °C. Suspended the purified ADSC-Exos with 100 µL PBS and stored at − 80 °C for further study. The protein extracted from the exosomes was quantified using the Bradford method. And then biomarker CD63 and CD9 were used to characterize the purified ADSC-Exos. The morphology of ADSC-Exo was detected through the transmission electron microscope (Hitachi, Tokyo, Japan). The size of ADSC-Exo was evaluated by Nanoparticle tracking analysis (NTA) analysis.

### Wound healing assay

Wound healing was used to measure proliferation, microvascular endothelial cells were plated at 3,000 cells/well in 96-well plates and treated with ADSC-Exos or vehicle for 3 days. Then the treated microvascular endothelial cells were seeded onto Culture Insert 2 Well in -Dish 35 mm (no. 81176, Ibidi). After administration, cells were cultured in serum-free medium overnight. Then removed the culture insert to create an ~ 500um cell-free gap, and covered the dish with culture medium. Cellular migration was visualized at the indicated time points. The width of the open area at each time point versus the width at time 0 was used to determine the extent of wound healing ratio.

### Assessment of apoptosis

TdT-Mediated dUTP Nick End-Labeling (TUNEL) Assay kit (In Situ Cell Death Detection Kit, Roche, CA, USA) was used to detect the apoptosis of cardiomyocytes in MI-treated mice. The percentage of TUNEL-positive nuclei relative to the total nucleus represents the apoptosis level of cardiomyocytes. In addition, the apoptotic microvascular endothelial cells were evaluated using flow cytometry.

### ADMSC oxidative damage measurement

Oxidative stress is characterized by the production of reactive oxygen species (ROS). They represent the injury of ADSCs in the setting of oxidation stress. The intracellular ROS production of ADSCs was assessed using 10 μM dihydroethidium (DHE; Invitrogen, San Diego, CA, USA). The ADSCs were treated with DHE, incubated in a dark environment, 37 °C and viewed using confocal laser microscopy (Olympus).

### Cell viability assessment

Cells were harvested 24 h after reoxygenation and incubated in a culture medium containing 10 μL of Cell Counting Kit-8 (CCK-8; Sigma Aldrich, Darmstadt, Germany) solution for another 2 h. Subsequently, the optical density was measured at a wavelength of 450 nm, and the cell proliferation rate was calculated.

### Statistical analysis

All data are presented as mean ± the standard error of the mean. Graphpad Prism 8.0 software was used for statistical analyses. For the analysis of two groups, unpaired two-tailed Student t-tests were conducted. When more than two groups were compared, one-way analysis of variance with post hoc analysis was performed. P values of 0.05 were considered statistically significant.

## Results

### Isolation and characterization of exosomes derived from ADSCs

**Fig. 1:**
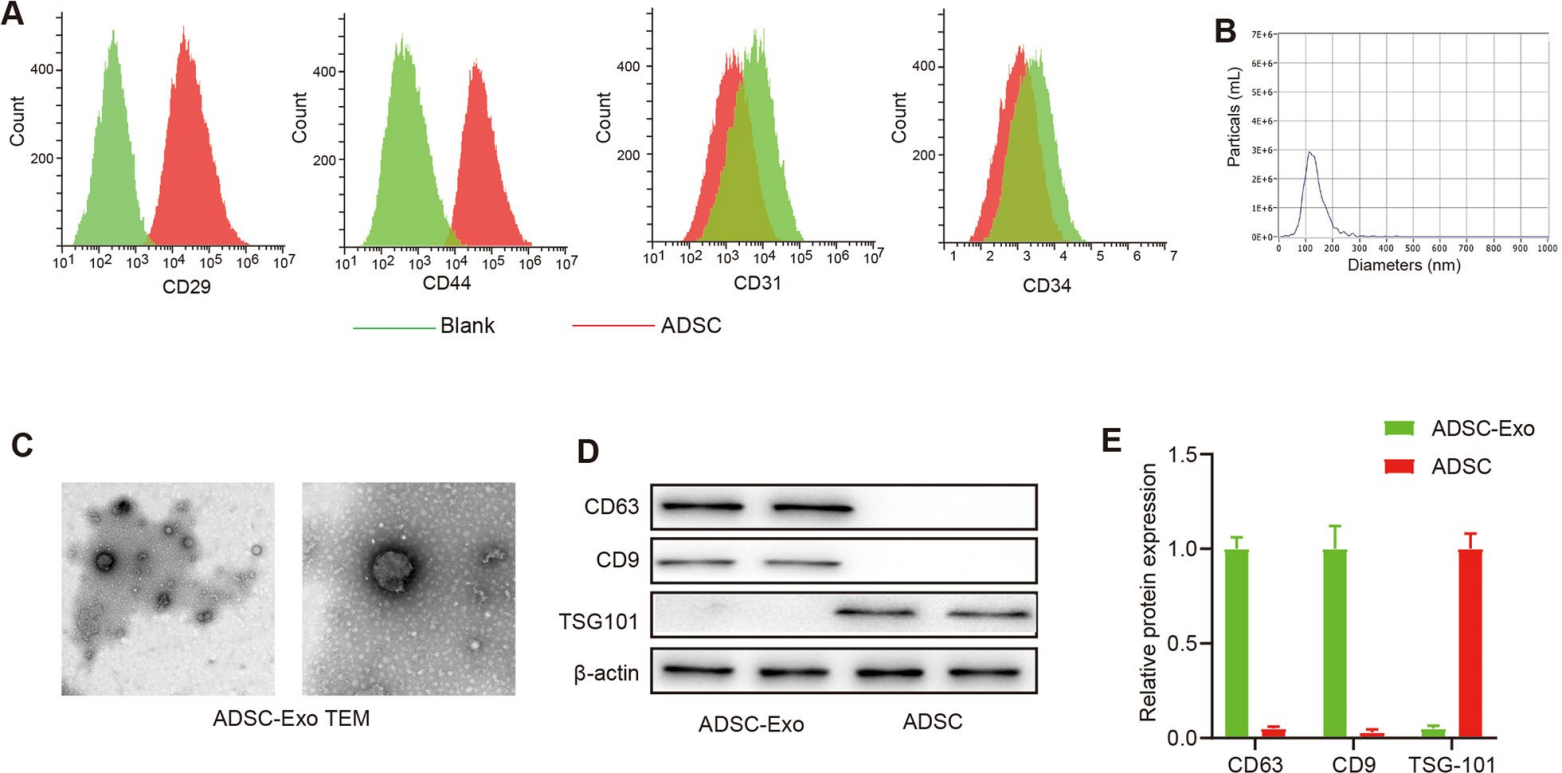
Isolation and characterization of exosomes derived from ADSCs **A.** The expression of relevant biomarkers of ADSCs (CD29, CD44, CD31 and CD34) were measured by flow cytometry; **B.** The concentration and size distribution of ADSC-Exo was detected by Nanoparticle tracking analysis (NTA); **C.** The morphology of ADSC-Exo was characterized by transmission electron microscope; **C**–**E** The relative expression of exosomes’ marker proteins (CD63, TSG101 and CD9) evaluated by Western blot;

ADSCs were isolated from the inguinal fat tissue of C57BL/6 wild-type mice. Passage 2 ADSCs were used in this experiment. We tested ADSCs using the expression levels of MSC surface marker such as CD29, CD31, CD34, and CD44 (Fig. 1A). The exosomes from ADSCs were observed as typical cup-shaped structures using transmission electron microscopy (TEM) (Fig. 1C). Nanoparticle Tracking Analysis (NTA) was performed to analyze the size distribution of exosomes [6]. They were approximately 100 nm in size (Fig. 1B). Furthermore, western blot analysis demonstrated that exosomes derived from ADSCs were positive for exosome-specific markers Alix, TSG101, CD81, and CD63 (Fig. 1D–E).

### Intravenous injection of ADSCs-Exos can reduce infarction area and improve post-MI cardiac function and remodeling

**Fig. 2:**
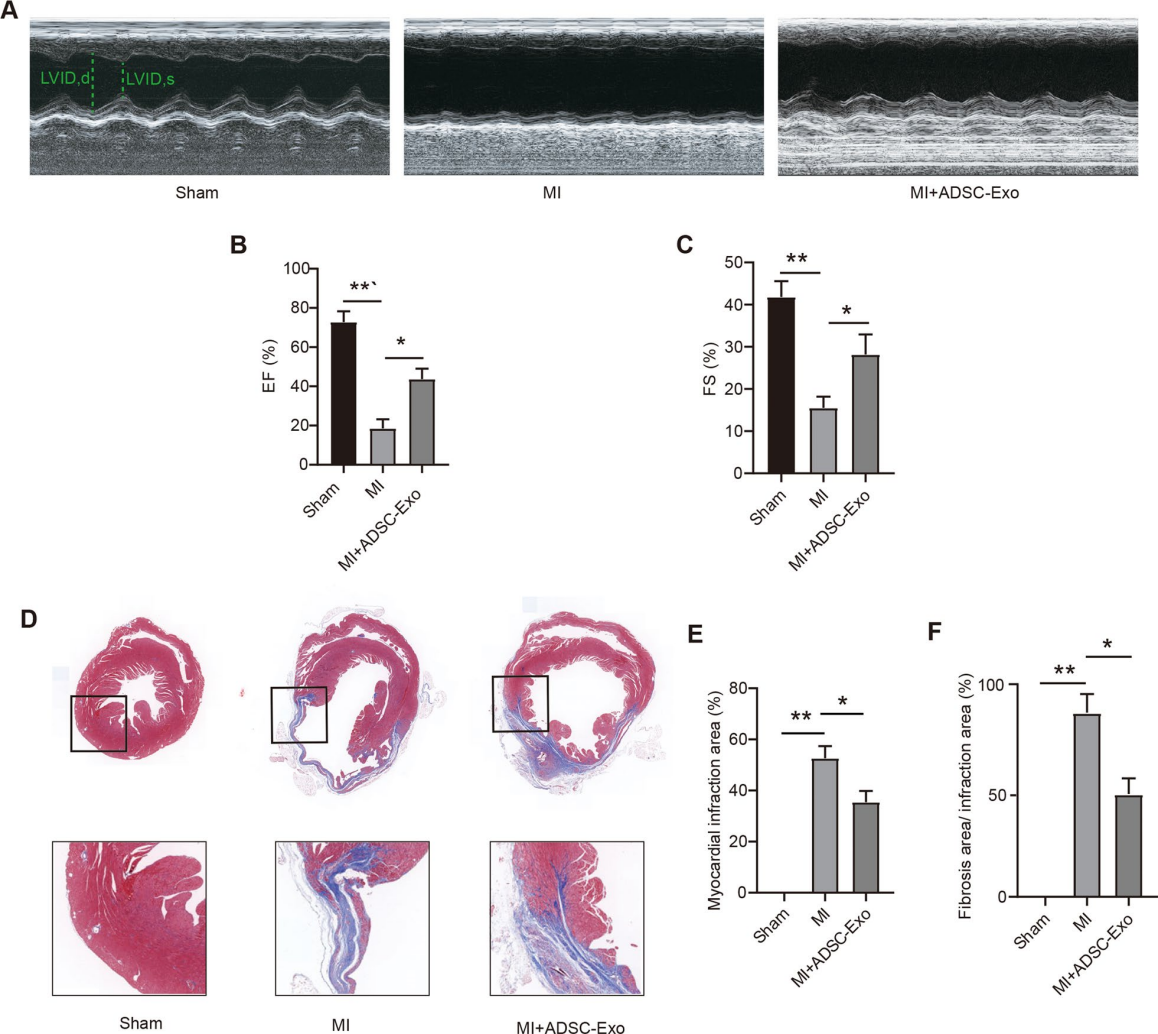
Intravenous injection of ADSCs-Exos can reduce infarction area and improve post-MI cardiac function and remodeling **A.** Echocardiography was used to evaluate cardiac function in control mice, MI-treated mice, and ADSC-Exo-treated MI mice; **B-C.** Representative analysis of left ventricular ejection fraction (EF) and fractional shortening (FS), compared with MI-treated mice, the EF and FS in the ADSC-Exo-treated MI mice were significantly increased; **D.** Masson staining for proportion of collagen in MI mice; **E–F.** Quantitative analysis of myocardial infarction area and the ration of fibrosis area and infarction area. Data were presented as Mean ± SEM, n = 8–10 mice. ^**^P < 0.05, ^*^P < 0.05

The infarcted myocardium can regenerate itself through excessive extracellular matrix (ECM) deposition, which replaces the dead cardiomyocytes and leads to scar formation. Myocardial fibrosis also occurs in most cardiac pathologic conditions and is associated with poor cardiac outcomes. Notably, myocardial fibrosis leads to increased cardiac remodeling and reduced ventricular compliance, all of which contribute to the progression of heart failure [21]. To investigate the beneficial role of exosomes in MI, purified ADSC-Exos were intramyocardially injected into the border zone of infarcted mice hearts after 25 min [6]. The echocardiography results showed that in MI mice, the ejection fraction (EF) and fractional shortening (FS) were significantly lower than in the sham group (Fig. 2A–C). In contrast to MI mice, ADSC-Exos administration improved the EF and FS (Fig. 2A–C). Moreover, the effects of ADSC-exo pretreated with miR-205 inhibitor in mice were evaluated. The results showed that compared with ADSC-Exo-treated MI mice, the left ventricular ejection fraction (EF) and fractional shortening (FS) in the ADSC-Exo + miR-205 inhibitor treated MI mice were significantly decreased (Additional file 1: Figure S1A-B). Masson trichrome staining showed that, compared with the sham group, the area of myocardial fibrosis in the MI group was significantly increased. Furthermore, the intravenous delivery of ADSC-Exos significantly reduced the infarct size and fibrosis area compared to the MI group (Fig. 2D-F). These results indicate that ADSC-Exos play a protective role in MI, to some extent, ADSC-Exo can prevent myocardial MI injury through miR-205 in vivo.

### Intravenous delivery of ADSCs-Exos promotes the survival of cardiomyocytes after MI

Studies have shown that acute and chronic loss of cardiomyocytes leads to pathological left ventricular remodeling and cardiac dysfunction, leading to the progression of heart failure [4]. It has been reported that the excessive production of ROS during MI and the ischemic environment increases cardiomyocytes apoptosis [22, [23]. Therefore, it is important to improve the ischemic or oxidative stress conditions to protect these cardiomyocytes from apoptosis [24].

**Fig. 3:**
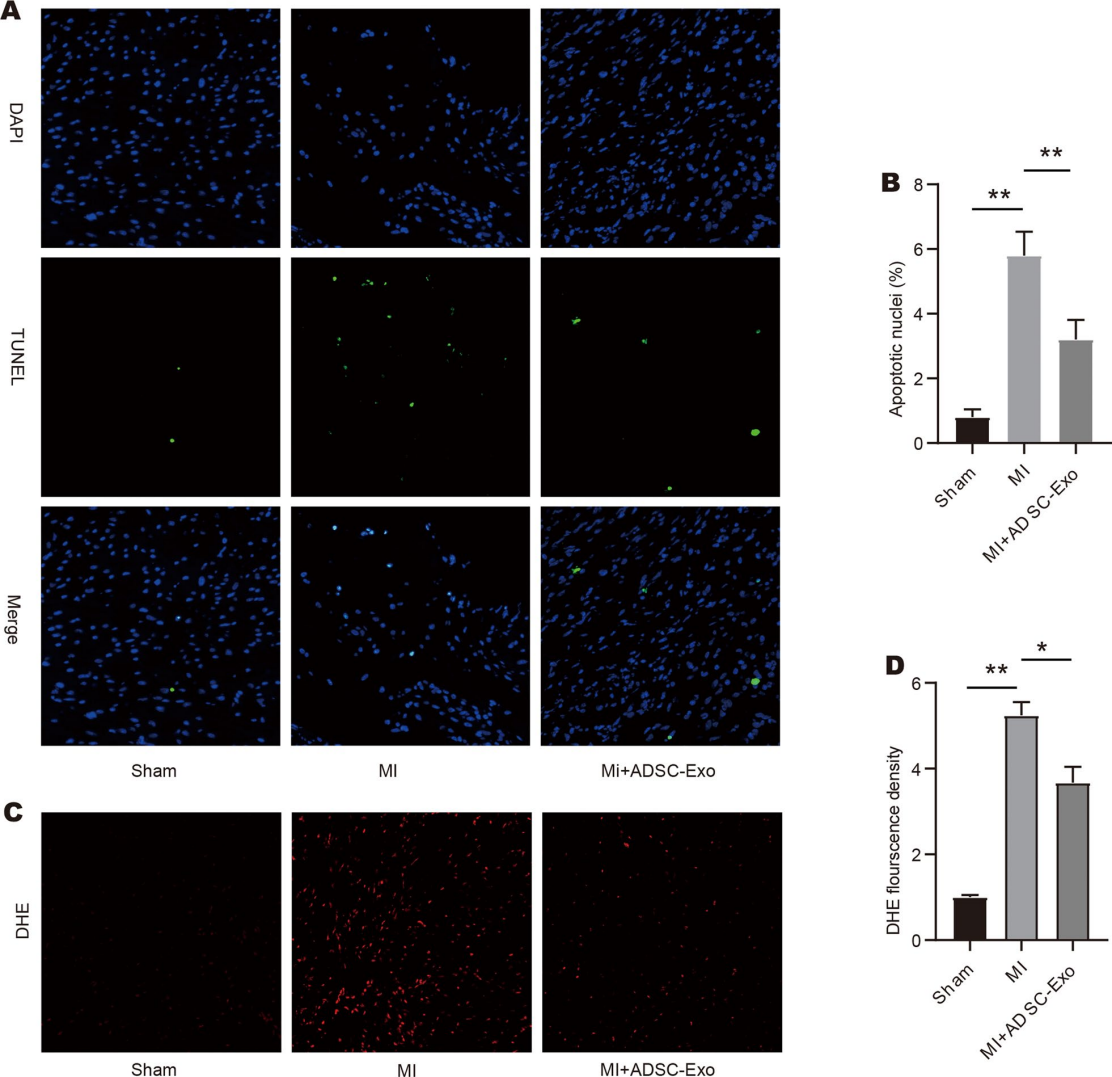
Intravenous delivery of ADSCs-Exos promotes the survival of cardiomyocytes after MI **A.** Representative apoptotic cardiomyocytes revealed by TUNEL staining; **B.** Quantitative analysis of the ratio of TUNEL-positive cardiomyocytes; **C-D.** The production of ROS in ischemic heart tissue was tested by DHE immunofluorescence staining. Data were presented as Mean ± SEM, n = 8–10 mice. ^**^P < 0.05, ^*^P < 0.05

Apoptosis of cardiomyocytes was evaluated using TUNEL staining. The number of TUNEL-positive cells in the MI areas was significantly higher than in the sham group (Fig. 3A–B). Compared with the MI group, the delivery of ADSC-Exos significantly reduced cardiomyocytes apoptosis (Fig. 3A–B). The production of ROS in ischemic heart tissue was tested using DHE immunofluorescence staining. The results showed marked red fluorescent protein (RFP^+^) fluorescence in the MI group. Consistent with previous reports, the group administered intravenously with ADSC-Exos had reduced RFP^+^ fluorescence compared with the MI group (Fig. 3C–D).

### Intravenous delivery of ADSCs-Exosomes promotes angiogenesis and microvascular endothelial cells proliferation

**Fig. 4:**
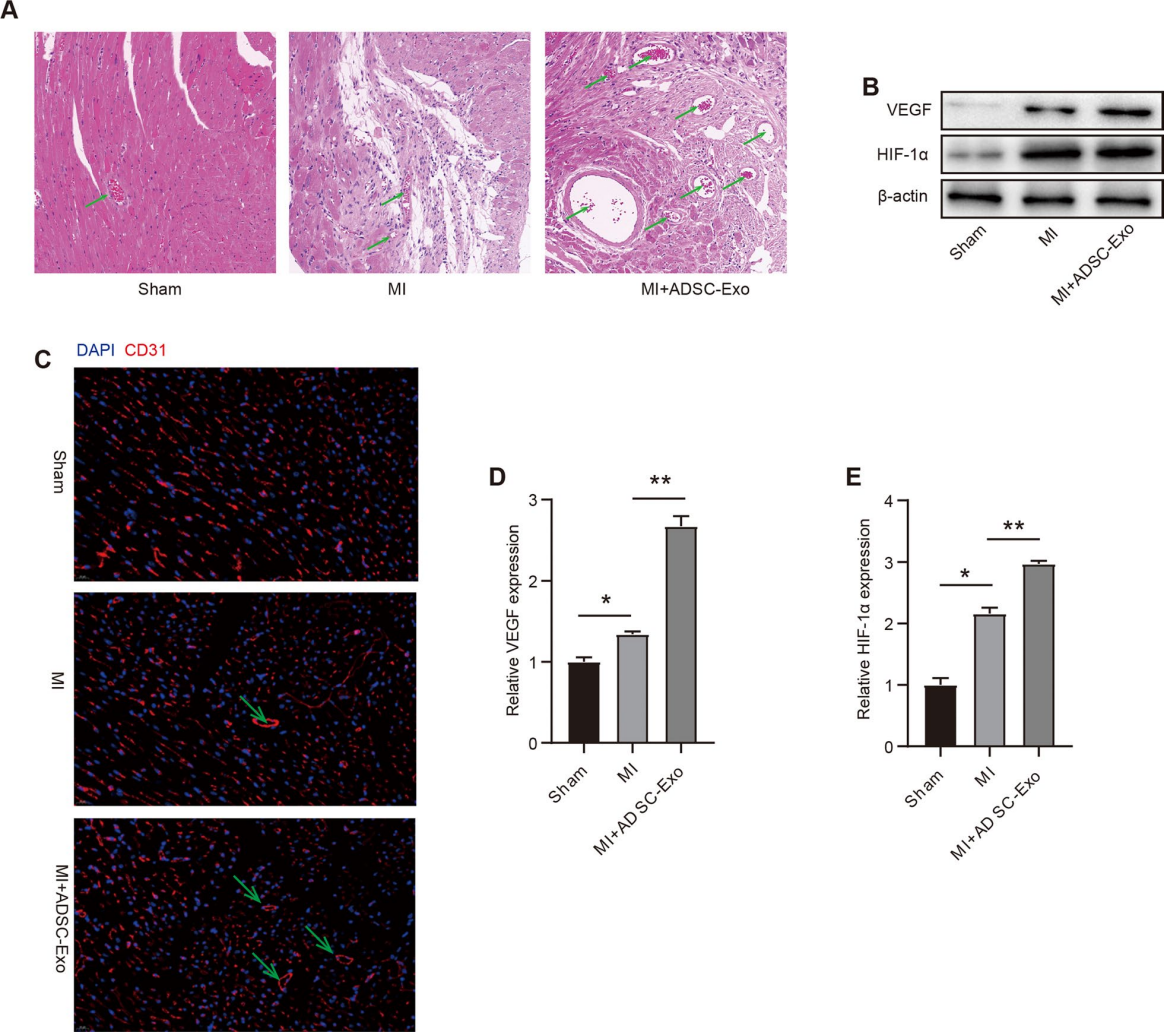
Intravenous delivery of ADSCs-Exosomes promotes angiogenesis and microvascular endothelial cells proliferation. **A.** Representative HE staining of neovessels in the hearts from the sham group, MI group and MI + ADSC-exo group; **B.** The expression of angiogenic marker HIF-1α and VEGF were evaluated by western blot; **C.** Fluorescent immunostaining of plaque sections with anti-CD31 antibody; **D-E.** Quantitative analysis of angiogenic marker HIF-1α and VEGF expression. Data were presented as Mean ± SEM, n = 8–10 mice. ^**^P < 0.05, ^*^P < 0.05

Angiogenesis has been reported to be a key component in the process of wound healing [25]. It is necessary for myocardial regeneration after MI [26]. The pathophysiology of MI involves the activation of hypoxia-inducible factor (HIF)-1α proteins and the release of vascular endothelial growth factor (VEGF) [27]. VEGF has also been shown to have angiogenic activity [28]. Various studies found that angiogenesis due to stem cell therapy preserved cardiac function following MI [29]. To explore the underlying protective mechanisms of ADSC-Exos in reducing infarct size, we assessed angiogenesis using CD31 immunofluorescence (Fig. 4A) and hematoxylin and eosin (HE) staining (Fig. 4C). The numbers of neovessels in the MI areas of hearts from the MI group did not differ significantly compared with the sham group (Fig. 4A and C). In contrast, the injection of ADSC-Exos significantly increased the number of neovessels compared with both sham and MI groups (both P < 0.01; Fig. 4A and C). Moreover, the expressions of activated angiogenic markers HIF-1α and VEGF were also evaluated using western blot analysis. The results showed that the expressions of HIF-1α and VEGF were higher in the MI group than in the sham group (Fig. 4B; D–E). Furthermore, compared with the MI group, the injection of ADSC-Exos significantly increased the expression of HIF-1α and VEGF (Fig. 4B; D–E). These results demonstrate that injection of ADSC-Exos increases angiogenesis, resulting in a cardioprotective role after MI.

### miRNA-205 was involved in the ADSC-Exos-mediated promotion of the proliferation of microvascular endothelial cells

**Fig. 5:**
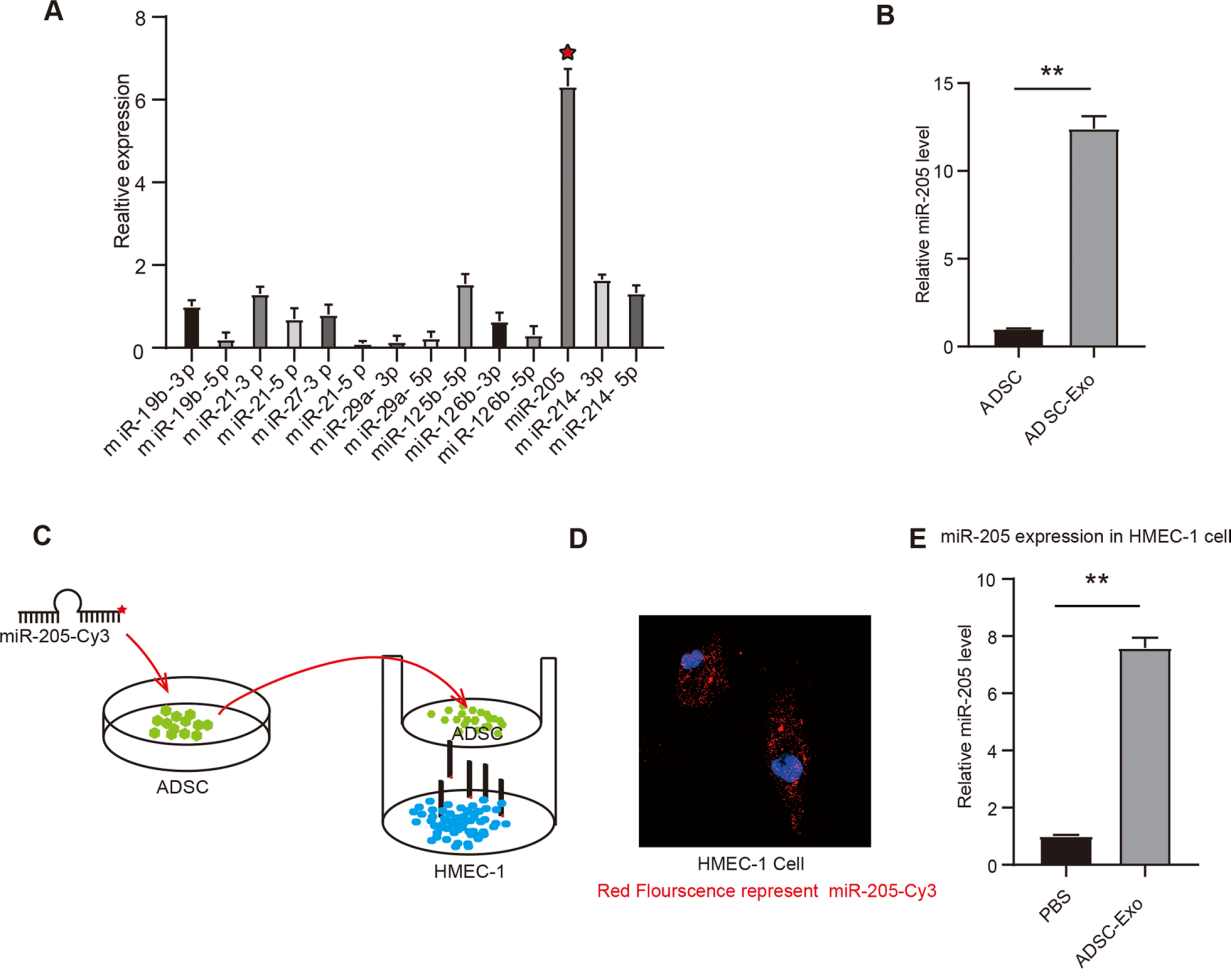
miRNA-205 was involved in the ADSC-Exos-mediated promotion of the proliferation of microvascular endothelial cells. **A.** The level of miR-205 in ADSCs-exosomes was evaluated by metabolomics analysis; **B.** The level of miR-205 in ADSCs-exosomes was determined by RT-qPCR; **C.** ADSC cells treated with cy3 labeled miR-205 were co-cultured with microvascular endothelial cells. **D.** Cy3 labeled miR-205 (red) were present in microvascular endothelial cells. **E.** Relative level of miR-205 in ADSCs-exosomes was determined by RT-qPCR. Data were presented as Mean ± SEM, n = 6 independent experiment. ^**^P < 0.05

ADSCs have been demonstrated to activate blood vessel formation, thus providing a promising future for therapeutic angiogenesis [30]. In recent angiogenesis studies, ADSCs have often been co-cultured with human microvascular endothelial cells (HMEC-1) to modulate endothelial cells and induce angiogenesis by promoting tube formation [31]. To identify the molecular mechanisms underlying the effects of ADSC-Exos on microvascular endothelial cell proliferation, metabolomics analysis was used to analyze miRNA levels in ADSC-Exos. The result showed that miRNA-205 was highly upregulated under an ischemic and hypoxic environment (Fig. 5A–B). To further confirm that the ADSCs and HMEC-1 communicate via miRNA-205, cy3-labeled miRNA-205 was added to the medium of ADSC cells, then they were co-cultured with microvascular endothelial cells (Fig. 5C). Immunofluorescence staining confirmed that HMEC-1 was labeled with miRNA-205-Cy3 (Fig. 5D). In addition, a negative control Cy3-labeled scrambled RNA (Cy3-ctrl) were also constructed and transfected to ADSC. Cy3 signal were successfully detected in ADSC cells (as shown in Additional file 2: Fig. S2A). Furthermore, we used quantitative real-time polymerase chain reaction to analyze miRNA-205 levels in ADSC-Exos-treated microvascular endothelial cells. The relative expression of miRNA-205 was upregulated more than twofold (Fig. 5E). These results suggest that miRNA-205 is involved in the ADSCs-Exos-mediated promotion of microvascular endothelial cell proliferation, thus promoting angiogenesis.

### Intravenously injected ADSCs-exosomes promoted hypoxia-treated HMEC-1 survival and angiogenesis through miR-205 after MI

**Fig. 6:**
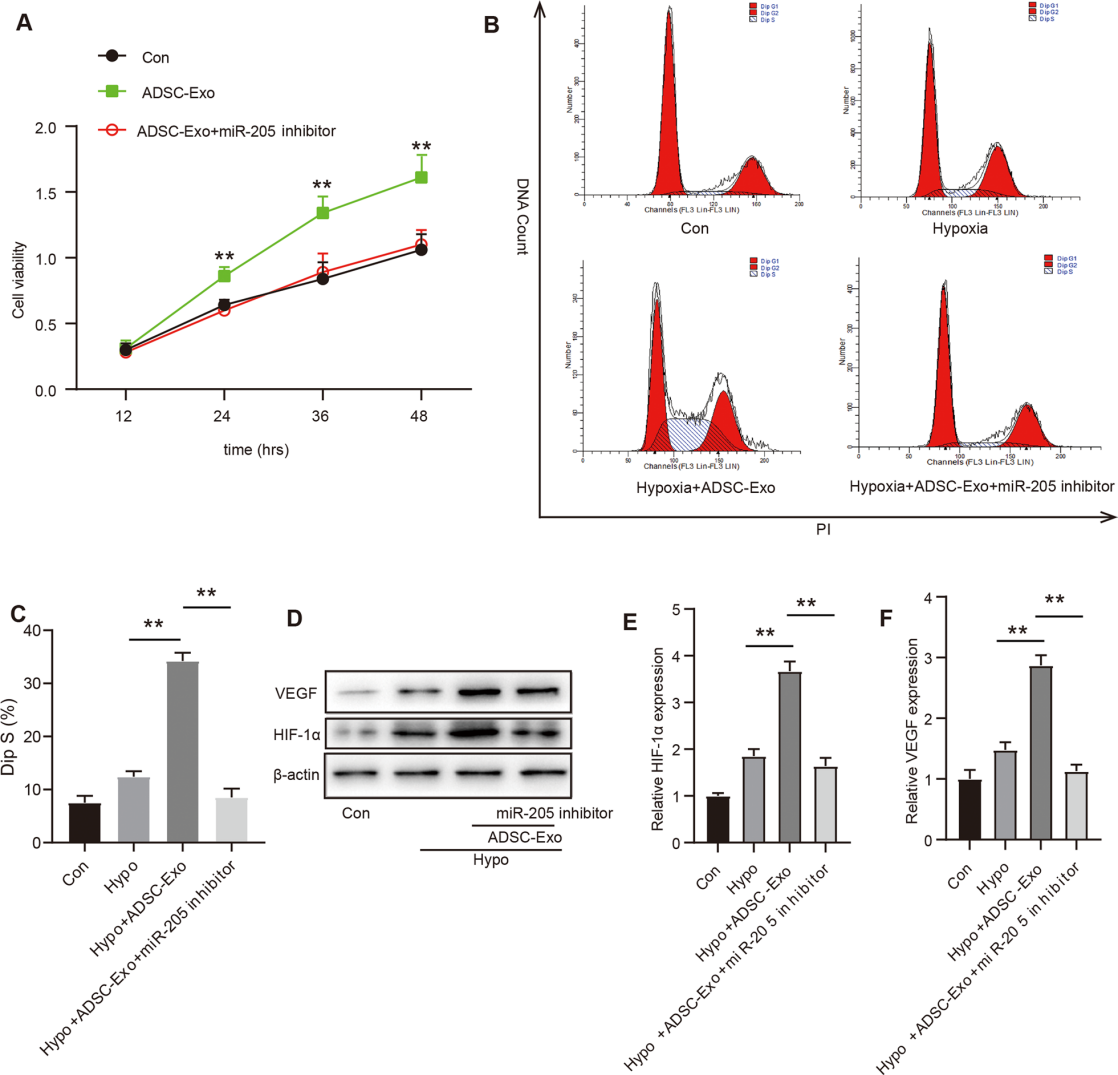
Intravenously injected ADSCs-exosomes promoted hypoxia-treated HMEC-1 survival and angiogenesis through miR-205 after MI. **A.** Effect of ADSC-Exo containing miR-205 on HMEC-1 viability after hypoxia injury was measured by MTT assay; **B.** Evaluation of HMEC-1 proliferation by flow cytometry is shown; **C.** Quantitative analysis of HMEC-1 proliferation; **D.** The level of angiogenic marker HIF-1α and VEGF were evaluated by Western blot analysis; **E–F.** Quantitative analysis of HIF-1α and VEGF expression. Data were presented as Mean ± SEM, n = 6 independent experiment. ^**^P < 0.05

To further investigate ADSC-Exos containing miR-205 play a critical role in HMEC-1 survival and the formation of neovessels. We performed a pretreatment of ADSCs with miR-205 mimics inhibitor. The result showed that ADSC-Exos markedly alleviated the impairment by hypoxia treatment, they promoted HMEC-1 survival (Fig. 6A). However, the protective effect of ADSC-Exos was significantly inhibited after treated with miR-205 mimics inhibitor (Fig. 6A). Similarly, flow cytometry assay also showed markedly reduced apoptosis of HMEC-1 with ADSC-Exos administration and critical increased apoptosis transfection with miR-205 mimics inhibitor (Fig. 6B–C). Additionally, activated angiogenic protein HIF-1α and VEGF were also measured by western blot (Fig. 6D). The result also confirmed that increased expression of HIF-1α and VEGF were found after adding ADSC-Exos, while transfection with miR-205 mimics inhibitor significantly decreased the expression of HIF-1α and VEGF (Fig. 6D–F). Taken together, these findings indicate that ADSCs-Exo containing miR-205 play a crucial role in promoting HMEC-1 survival and angiogenesis in MI.

### Intravenously injected ADSCs-exosomes prevented the apoptotic rate of hypoxia-treated HMEC-1 cells through miR-205 after MI

**Fig. 7:**
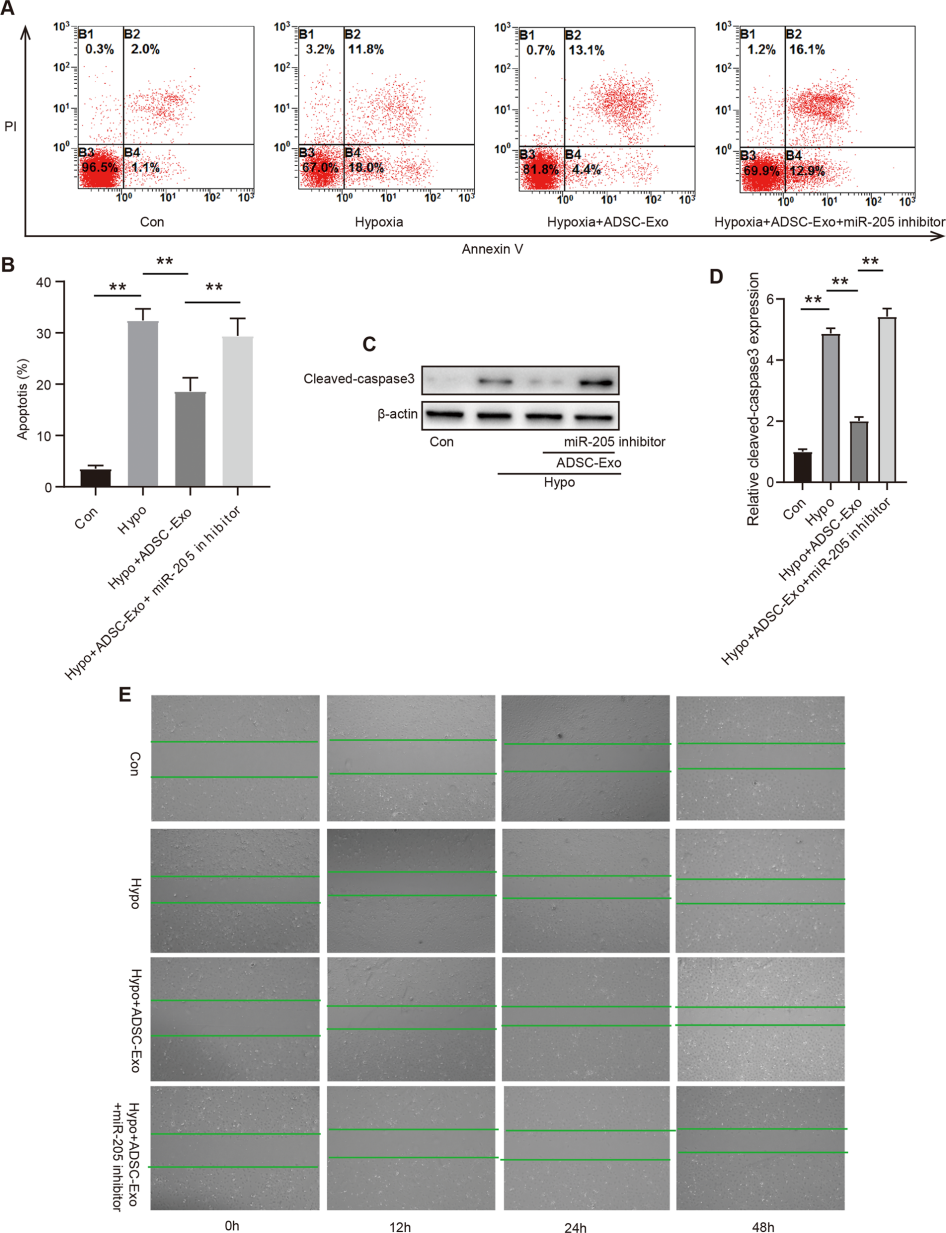
Intravenously injected ADSCs-exosomes prevented the apoptotic rate of hypoxia-treated HMEC-1 cells through miR-205 after MI. **A.** The evaluation of HMEC-1 apoptosis was measured by flow cytometry; **B.** Quantitative analysis of the apoptotic ratio of HMEC-1 by flow cytometry; **C.** The level of caspase-3 was evaluated by Western blot analysis; **D.** Quantitative analysis of caspase-3 expression. **E.** Effect of ADSC-Exo containing miR-205 on microvascular endothelial cells migration was detected by Wound-Healing Assay. Mean ± SEM, n = 6 independent experiment. ^**^P < 0.05

To further investigate the protective mechanism of ADSC-Exos against myocardial ischemic injury, a miRNA-205 inhibitor was used to confirm the effects of miRNA-205 on HMEC-1 cells apoptosis. HMEC-1 cells were subjected to hypoxia treatment for 2 h to mimic myocardial ischemic injury in vitro. The levels of apoptosis in cells were evaluated using flow cytometry (Fig. 7A–B). The result showed that transfection with ADSC-Exos significantly reduced the apoptotic rate of hypoxia-treated HMEC-1 compared to that in the control group (Fig. 7A–B). However, the miRNA-205 inhibitor significantly increased the level of apoptosis in HMEC-1 (Fig. 7A–B). In addition, the expression levels of apoptotic proteins such as caspase-3 were measured (Fig. 7C). Western blot analysis indicated that hypoxia treatment significantly increased the levels of caspase-3, and transfection with miRNA-205 inhibitor further promoted the expression of caspase-3 (Fig. 7C–D). However, ADSC-Exos markedly reversed the increase in the level of caspase-3 (Fig. 7C–D). Furthermore, in order to elucidate the protective effects of miR-205 on cardiomyocytes, the level of apoptotic neonatal cardiomyocytes in vitro undergoing hypoxia were evaluated by flow cytometry. However, the result showed that ADSC-exo with and without miR-205 inhibitor had no protective effect on neonatal cardiomyocytes apoptosis undergoing hypoxia environment (Additional file 3: Fig. S3A–B). These results of the scratch experiment showed that ADSC-Exos improved the migratory ability of HMEC-1, which was also blunted by the miRNA-205 inhibitor (Fig. 7E). These findings indicate that ADSC-Exos protect against hypoxia-induced HMEC-1 cells apoptosis and promote HMEC-1 cells migration via miRNA-205.

## Discussion

The incidence of AMI has been reported to have increased rapidly in China in recent decades [1]. Although timely thrombolysis and percutaneous coronary intervention (PCI) are effective therapeutic strategies for patients with MI in terms of reducing the size of the infarcted area and myocardial ischemic injury [32], it remains the leading cause of global mortality [33]. Massive cardiomyocytes loss due to increased oxidative stress, inflammation, and induced myocardial apoptosis is still irreversible [6]. Various studies found that ADSCs are potential strategies for the treatment of ischemic heart disease [2]. However, the intravenous injection of ADSCs resulted in limited myocardial stem cell retention and survival [8, [34]. In this study, we found that ADSC-Exos decreased myocardial apoptosis and increased angiogenesis, thus contributing to cardiac function recovery after MI. We have also demonstrated that the cardioprotective effects of ADSC-Exos are achieved through large amounts of miRNA-205.

Since cardiomyocytes have little regenerative ability, reducing the apoptosis of cardiomyocytes after an injury has a promising therapeutic potential [35]. ADSCs are a type of MSC that can be easily obtained from a stromal vascular fraction (SVF) within adipose tissues [36]. ADSCs differ from other MSCs in that they are more readily available, have a high proliferation potential, have extraordinary self-renewal ability, and can secrete nutritional factors and extracellular vesicles, making them the ideal treatment candidate for cardiac function recovery [37]. Various studies have indicated that ADSCs play a central role in chemoattractant and promote angiogenesis through VEGF expression, thus contributing to cardiac tissue regeneration [38, [39]. Other studies have also indicated ADSCs can secrete many anti‐apoptotic, pro‐angiogenic, and anti‐inflammatory cytokines and growth factors, thus contributing to inhibiting adverse cardiac remodeling and improving ventricular function and myocardial vascularization in the infarcted myocardium [8]. Although the intravenous administration of ADSCs immediately showed better benefits after the reperfusion, the intravenous delivery method, the ideal concentration, and timing for cell administration remain unexamined [8]. Furthermore, the beneficial effects of stem cells were affected due to the poor survival, retention, and engraftment of MSCs following transplantation [40]. Although there are various invasive strategies to deliver ADSCs such as catheter-based delivery (intracoronary or trans-endocardial injection) or surgical delivery (direct intramyocardial injection) in injured myocardium, the retention of ADSCs was limited, and abundant ADSCs were distributed to the lungs [7, [8, [34]. As a result, it is critical to address the issue of ADSC redistribution to the lungs and improve the limited cardiac ADSC retention. Exosome-containing short RNAs released by MSCs can regulate the microenvironment in stem cells, which is dependent on the balance between differentiation and proliferation of stem cells [41]. These findings could help shed light on the regulatory mechanism that controls MSC paracrine activity, which is responsible for the tissue-specific regenerative characteristics of MSCs [41]. In this study, MI injury exacerbated cardiac dysfunction and promoted cardiac fibrosis. Furthermore, MI surgery significantly increased cardiomyocytes apoptosis, while ADSC-Exos administration markedly reversed these effects. ADSC-Exos reduces cardiomyocytes loss by inhibiting myocardial apoptosis and can be beneficial for MI-induced damage.

In recent years, exosomes have attracted much attention and they are a type of extracellular vesicle that range from 30 to 100 nm [42]. Exosomes contain biomolecules such as proteins, nucleic acids (DNA, mRNA, miRNA), lipids, and enzymes. They play important roles in cell-to-cell communication by transporting these biomolecules across various cells [42]. Adipose tissue has been demonstrated to play a central role in wound healing and tissue regeneration. Both ADSCs and ADSC-Exos are very important derivatives of fat tissues [42]. Researchers have found that although ADSC-Exos have no differentiation ability, they can mimic the capacity of ADSCs by lowering injury and inflammation damage, making them a suitable therapeutic target for damaged tissue regeneration and repair [42]. Furthermore, ADSC-Exos are small non-living substances with production and delivery advantages, making them a promising candidate for biological products. Most importantly, ADSC-Exos may be a safer therapeutic agent than ADSCs [42]. Additionally, the use of ADSC-Exos may effectively eliminate the issues that come with ADSC administration, such as limited ADSC survival, intensive immune rejection, functional inactivation, and unfavorable differentiation [43]. ADSC-Exos have been shown to offer therapeutic potential for targeted drug delivery and to be a promising candidate for regenerative medicine in skin healing and various reconstructive operations [44]. According to research, exosomes play an important role in immune response, antigen presentation, tumor cell migration and proliferation, apoptosis, and autophagy, thus they participate in the pathophysiology of many diseases [12]. When the homeostasis of the microenvironment of the tissue is disrupted by harmful stimuli such as disease or injury, MSC-derived exosomes play an important role in homeostasis [14]. We found that ADSC-Exos can inhibit cardiomyocyte apoptosis and promote angiogenesis, thus contributing to improving cardiac function in MI-treated mice. Based on these findings, ADSC-Exos has therapeutic potential to improve myocardial function.

Emerging evidence suggests that miRNAs play an important role in information transferring between cells [14]. Exosomal miRNAs are the most abundant molecules found in exosomes [12]. It is increasingly considered that miRNAs contribute to increased self-renewal of stem cells, and promote their differentiation and pluripotency [45]. Additionally, miRNAs play a central role in regulating the proliferation, differentiation, and survival of MSCs [46]. Exosomes as information carriers are thought to play a key role in miRNA-mediated cell-to-cell communication, according to growing evidence [14]. The diverse functions of exosomal miRNAs were observed in many physiological and pathological processes, such as inflammation, cell migration, proliferation, apoptosis, autophagy, and epithelial-mesenchymal transition [12]. There is increasing evidence showing that dysregulation of exosomal miRNAs occurs in various pathophysiological processes including atherosclerosis, acute coronary syndrome, heart failure, myocardial ischemia–reperfusion injury, and pulmonary hypertension [12, [47]. In these studies, miRNA-205 was demonstrated to be involved in the induction of inflammation and atherosclerosis in vascular endothelial cells by targeting the tissue inhibitor of metalloproteinase-3, which interferes with miRNA-205 expression and thus plays a protective role in vascular endothelial cells [18]. Studies have shown that the expression level of miRNA-205 is associated with the inhibition of apoptosis [17]. It has also been suggested that MI and cardiomyopathy can aggravate myocardial apoptosis and contribute to the progression of heart failure. Therefore, miRNA-205 has therapeutic potential to alleviate myocardial damage. In our study, we demonstrated that exosomes containing miRNA-205 can regulate myocardial apoptosis, ameliorate MI injury, and improve cardiac function. We also found that exosomes derived from ADSCs containing miRNA-205 can promote the proliferation and migration of microvascular endothelial cells, facilitate angiogenesis, and inhibit cardiomyocyte apoptosis. We demonstrated that ADSC-Exos containing miRNA-205 is a key mediator between ADSCs and microvascular endothelial cells, thereby regulating cardiac function recovery. Based on these findings, we concluded that ADSC-Exos containing miRNA205 have promising therapeutic potential in MI-induced heart injury.

## Conclusion

In summary, we comprehensively investigated the functional role and molecular mechanisms of ADSC-Exos containing miRNA-205 in MI injury. Our findings suggest that ADSC-Exos containing miRNA-205 reduces myocardial fibrosis and inhibits myocardial apoptosis, both of which are important for restoring cardiac function in mice with MI injury. In addition, we also found that ADSC-Exos containing miRNA-205 can promote angiogenesis. Therefore, the results of the present study provide basic evidence for the application of ADSC-Exos in clinical treatments for MI.

## Supplementary Information

**Additional file 1**: **Figure 1** Intravenous injection of ADSCs-Exos pretreated with miR-205 inhibitor can aggravate cardiac function in post-MI mice A. Echocardiography was used to evaluate cardiac function in ADSC-Exo-treated MI mice and ADSC-Exo+miR-205 inhibitor treated MI mice; B. Representative analysis of left ventricular ejection fraction (EF) and fractional shortening (FS), compared with ADSC-Exo-treated MI mice, the EF and FS in the ADSC-Exo+miR-205 inhibitor treated MI mice were significantly decreased. Data were presented as Mean± SEM, n=8-10 mice. *P<0.05.

**Additional file 2**: **Figure 2** Cy3-labelled miRNA205 are kept by endothelial cells

**Additional file 3**: **Figure 3** ADSC-exo with and without miR-205 inhibitor has no effect on the apoptosis of neonatal cardiomyocytes A. Representative apoptotic neonatal cardiomyocytes revealed by Flow cytometry; B. Quantitative analysis of the ratio of apoptotic cardiomyocytes. Data were presented as Mean± SEM, n=6 independent experiment. *P<0.05.

## Data Availability

The datasets used and/or analyzed during the present study are available from the corresponding author on reasonable request.

## Abbreviations

MI: Acute myocardial infarction
ADSC-Exos: Adipose tissue-derived mesenchymal stem cell
MiRNA-205: MicroRNA205
HMEC-1: Human microvascular endothelial cell line
AMI: Acute myocardial infarction
MSCs: Mesenchymal stem cells
DNA: Deoxyribonucleic acid
mRNAs: Messenger RNA
MiRNAs: MicroRNAs
PBS: Phosphate-buffered saline
FBS: Fetal bovine serum
DMEM: Dulbecco’s modified eagle medium
HIF-1α: Hypoxia-inducible factor
VEGF: Vascular endothelial growth factor
HE: Hematoxylin and eosin
ROS: Reactive oxygen species
