# Supplementary figures and images for: ADSC-derived exosomes attenuate myocardial infarction injury by promoting miR-205-mediated cardiac angiogenesis
Source: Biol Direct. 2023 Feb 27;18:6. doi: 10.1186/s13062-023-00361-1 (PMC9972746; doi:10.1186/s13062-023-00361-1)

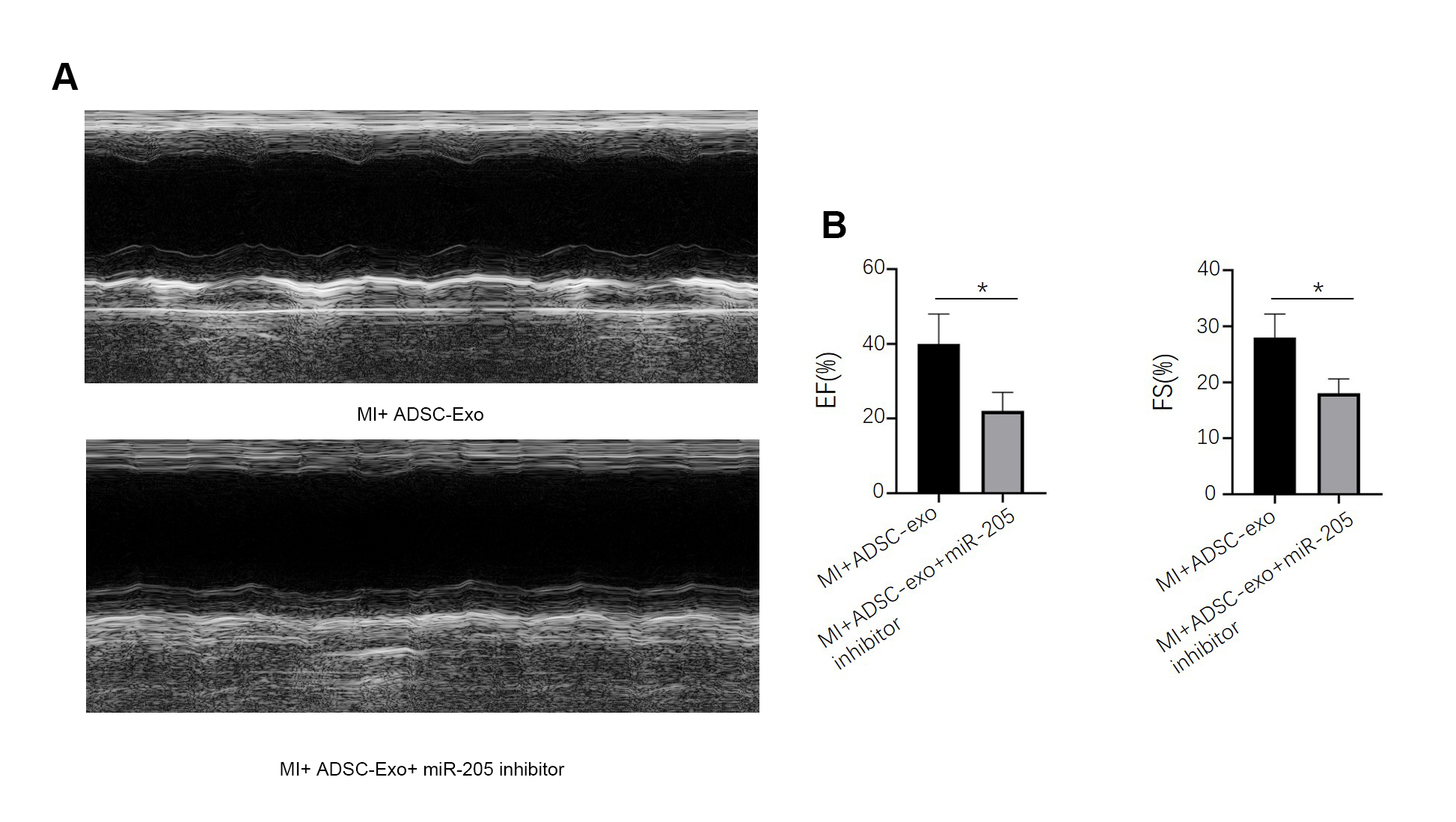

Supplement: Supplementary file 1 — Additional file 1: Figure 1 Intravenous injection of ADSCs-Exos pretreated with miR-205 inhibitor can aggravate cardiac function in post-MI mice A. Echocardiography was used to evaluate cardiac function in ADSC-Exo-treated MI mice and ADSC-Exo+miR-205 inhibitor treated MI mice; B. Representative analysis of left ventricular ejection fraction (EF) and fractional shortening (FS), compared with ADSC-Exo-treated MI mice, the EF and FS in the ADSC-Exo+miR-205 inhibitor treated MI mice were significantly decreased. Data were presented as Mean± SEM, n=8-10 mice. *P<0.05. [file 13062_2023_361_MOESM1_ESM.jpg]

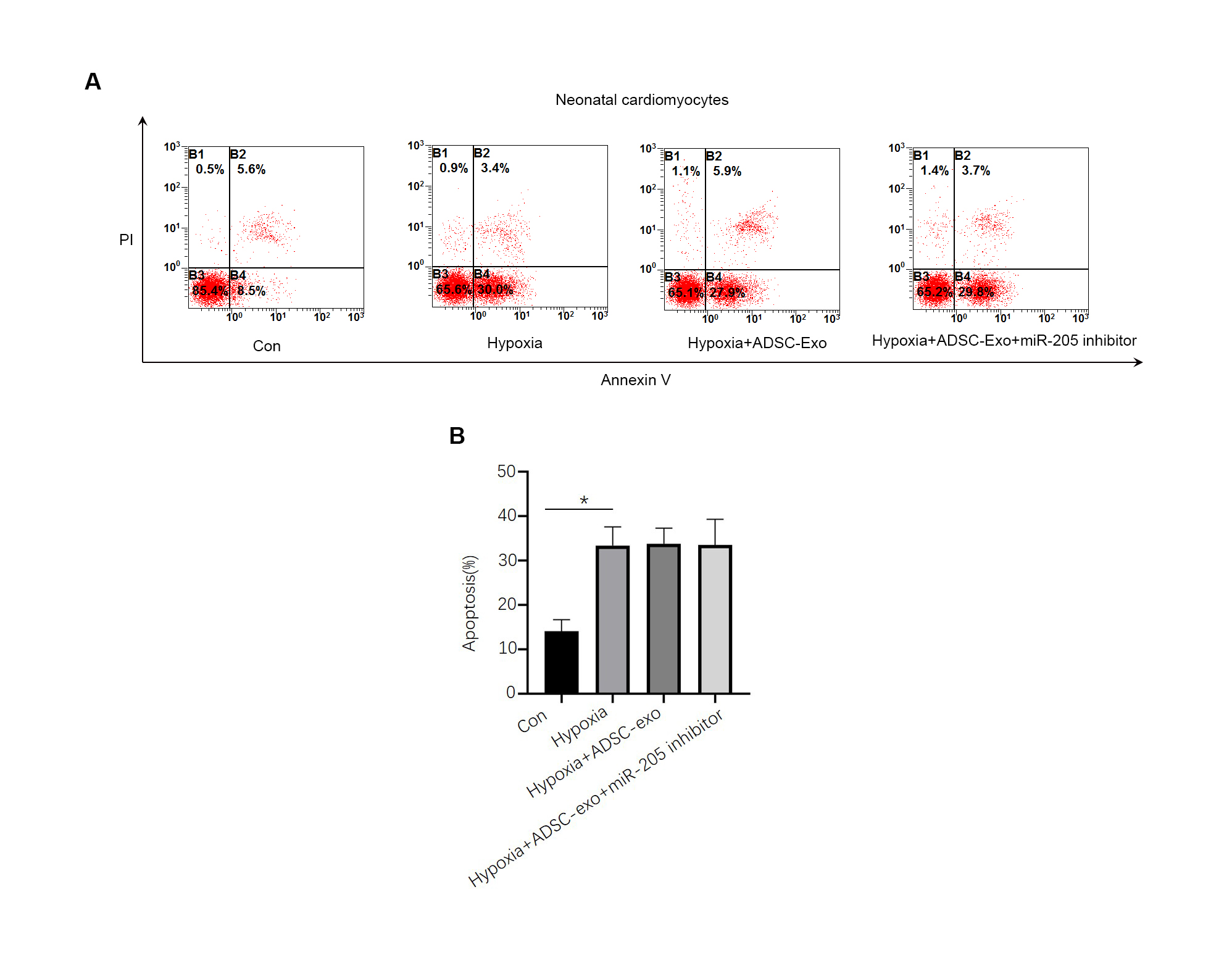

Supplement: Supplementary file 2 — Additional file 2: Figure 2 Cy3-labelled miRNA205 are kept by endothelial cells [file 13062_2023_361_MOESM2_ESM.jpg]

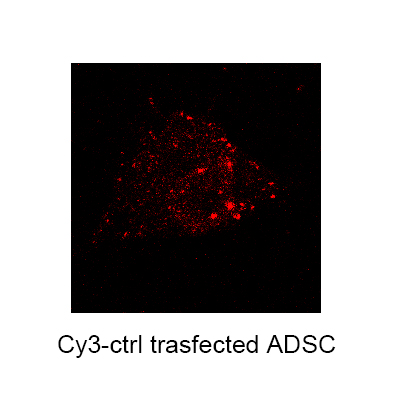

Supplement: Supplementary file 3 — Additional file 3: Figure 3 ADSC-exo with and without miR-205 inhibitor has no effect on the apoptosis of neonatal cardiomyocytes A. Representative apoptotic neonatal cardiomyocytes revealed by Flow cytometry; B. Quantitative analysis of the ratio of apoptotic cardiomyocytes. Data were presented as Mean± SEM, n=6 independent experiment. *P<0.05. [file 13062_2023_361_MOESM3_ESM.jpg]
